# Cardiometabolic Risk Factors in Childhood, Adolescent and Young Adult Survivors of Acute Lymphoblastic Leukemia – A Petale Cohort

**DOI:** 10.1038/s41598-017-17716-0

**Published:** 2017-12-15

**Authors:** Emile Levy, Mariia Samoilenko, Sophia Morel, Jade England, Devendra Amre, Laurence Bertout, Simon Drouin, Caroline Laverdière, Maja Krajinovic, Daniel Sinnett, Geneviève Lefebvre, Valérie Marcil

**Affiliations:** 1 Research Centre of Sainte-Justine University Health Center, Université de Montréal, Montréal, Quebec H3T 1C5 Canada; 2 Department of Nutrition, Université de Montréal, Montréal, Quebec H3T 1C5 Canada; 3 Department of Mathematics, Université du Québec à Montréal, Montréal, Quebec H3C 3P8 Canada; 4 Department of Pediatrics, Université de Montréal, Montréal, Quebec H3T 1C5 Canada

## Abstract

Our objectives were to assess the prevalence of cardiometabolic complications in children, adolescents, and young adult survivors of childhood acute lymphoblastic leukemia (cALL), to identify their predictors and the risk compared to the Canadian population. We performed a cardiometabolic assessment of cALL survivors from the PETALE cohort (n = 247, median age at visit of 21.7 years). In our group, overweight and obesity affected over 70% of women. Pre-hypertension and hypertension were mostly common in men, both adults (20%) and children (19%). Prediabetes was mainly present in women (6.1% of female adult survivors) and 41.3% had dyslipidemia. Cranial radiation therapy was a predictor of dyslipidemia (RR: 1.60, 95% CI: 1.07–2.41) and high LDL-cholesterol (RR: 4.78, 95% CI: 1.72–13.28). Male gender was a predictor for pre-hypertension and hypertension (RR: 5.12, 95% CI: 1.81–14.46). Obesity at the end of treatment was a predictor of obesity at interview (RR: 2.07, 95% CI: 1.37–3.14) and of metabolic syndrome (RR: 3.04, 95% CI: 1.14–8.09). Compared to the general population, cALL survivors were at higher risk of having the metabolic syndrome, dyslipidemia, pre-hypertension/hypertension and high LDL-cholesterol, while the risk for obesity was not different. Our results support the need for early screening and lifestyle intervention in this population.

## Introduction

Childhood acute lymphoblastic leukemia (cALL) is the most common pediatric malignancy accounting for almost one-third of all childhood cancers. With the advent of multimodality therapy, the long-term survival rate of children with ALL has remarkably improved and now exceeds 80%^1^. Nevertheless, for many patients the price for this success is very high since a majority of survivors will be afflicted by long-term treatment-related sequelae. Indeed, survivors of cALL are at increased risk for long-term cardiovascular complications, with a higher cardiac standardized mortality ratio of 3.8 compared with the US population^2^, and a 6.9-fold greater likelihood of developing cardiac complications when compared with a group of siblings^3^. Furthermore, studies have reported that survivors of cALL are at increased risk of metabolic syndrome characterized by obesity, insulin resistance, dyslipidemia and hypertension^4–6^, aortic calcifications^7^ and arterial changes^8^ that are suggestive of premature atherosclerosis and cardiovascular disease. Even during childhood, cardiovascular risk factors, including adiposity, physical inactivity, dyslipidemia and insulin resistance, have been identified in survivors^9^. The reported prevalence of metabolic syndrome in adults surviving cALL ranges between 6.9 to 39%^6,10–13^. This wide range can be explained by the heterogeneity and inconsistency among studies as some were conducted in non-homogeneous populations, only assessed certain risk factors, or included patients who received treatment regimens encompassing multiple decades.

The high prevalence of obesity and cardiometabolic complications in cALL survivors is not fully understood. Cranial radiotherapy (CRT) was found associated with elevated fat mass, hyperleptinemia, and impaired insulin sensitivity^14^. Since treatment protocols are now trending towards less use and lower doses of CRT, one could ask whether patients treated with modern therapy are still at risk of long-term cardiometabolic complications^15^. While CRT in doses of 20 Gray (Gy) or more was identified as the primary risk factor for obesity^16^, doses between 10 and 20 Gy used in more recent protocols were also associated with an increased risk^16^ indicating that survivors are still at risk despite lower CRT exposition. Other possible risk factors for obesity in cALL survivors include age and body mass index (BMI) at diagnosis^17–19^. Furthermore, the age of onset of cardiometabolic complications in cALL survivors remains nebulous since most studies have dealt with adult patients, with little data on childhood, adolescent and young adult (CAYA) survivors. Finally, few comprehensive comparisons to cohorts of national data have been carried out, especially in CAYA.

## Results

### Patient characteristics

Demographic and treatment characteristics relevant for the following analyses are outlined in Table 1. A total of 247 cALL survivors were included in this study (49.4% male). Median age at PETALE (*Prévenir les effets tardifs des traitements de la leucémie aigüe lymphoblastique chez l’enfant*) interview was 21.7 years old, ranging from 8.5 to 41.0 years (34.4% children), and median survival time was 15.2 years. A total of 147 participants (59.5%) received CRT. Given the reduction of CRT use in more recent protocols, a larger proportion of adult survivors received CRT as opposed to pediatric survivors (69.8 vs. 40.0%). For all protocols, doses of CRT ranged between 10 and 20 Gy.

**Table 1:** Demographic and treatment characteristics of survivors of childhood acute lymphoblastic leukemia from the PETALE cohort.

Characteristic	Total (N = 247)	PETALE	
		Adults (N = 162)	Children (N = 85)
Gender, N (%)			
Male	122 (49.4)	80 (49.4)	42 (49.4)
Female	125 (50.6)	82 (50.6)	43 (50.6)
Age at interview, years			
Mean (SD)	22.1 (6.3)	25.5 (5.1)	15.7 (1.8)
Median (range)	21.7 (8.5–41.0)	24.7 (18.0–41.0)	16.2 (8.5–17.9)
Age at cancer diagnosis, y			
Mean (SD)	6.6 (4.6)	7.9 (5.0)	4.2 (2.2)
Median (range)	4.7 (0.8–18.0)	6.5 (0.8–18.0)	3.6 (1.2–11.0)
Time from diagnosis, y			
Mean (SD)	15.5 (5.2)	17.7 (5.0)	11.5 (2.5)
Median (range)	15.2 (5.4–28.2)	18.3 (6.2–28.2)	11.9 (5.4–15.6)
Risk groups, N (%)			
Standard risk	113 (45.8)	59 (36.4)	54 (63.5)
High risk	128 (51.8)	100 (61.7)	28 (32.9)
Very high risk	6 (2.4)	3 (1.9)	3 (3.53)
Treatment protocol, N (%)			
DFCI 87–01	24 (9.7)	24 (14.8)	0 (0.0)
DFCI 91–01	46 (18.6)	46 (28.4)	0 (0.0)
DFCI 95–01	73 (29.6)	56 (34.6)	17 (20.0)
DFCI 2000–01	77 (31.2)	21 (13.0)	56 (65.9)
DFCI 2005–01	27 (10.9)	15 (9.3)	12 (14.1)
CRT exposure, N (%)			
Total	147 (59.5)	113 (69.8)	34 (40.0)
DFCI 87–01	18 (7.3)	18 (11.1)	0 (0.0)
DFCI 91–01	37 (15.0)	37 (22.8)	0 (0.0)
DFCI 95–01	45 (18.2)	34 (21.0)	11 (12.9)
DFCI 2000–01	33 (13.4)	17 (10.5)	16 (18.8)
DFCI 2005–01	14 (5.7)	7 (4.3)	7 (8.2)

CRT, cranial radiation therapy; DFCI, Dana Farber Cancer Institute. CRT doses were ranging between 10 and 20 gray.

### Prevalence of cardiometabolic complications in the PETALE cohort

The prevalence of cardiometabolic complications and the distribution of outcomes according to cut-off values are presented in Table 2 and in Supplementary Table 2. Data show that overweight and obesity were highly prevalent, particularly in women (affecting over 70% of them). Pre- and hypertension was mostly common in men (20%) and in boys (19%). Prediabetes was mainly present in women (prevalence of 6.1%). Importantly, we found a very high prevalence of dyslipidemia in all groups, as 41.3% of patients presented with one or more abnormal lipid values. In particular, we found low HDL-cholesterol (high-density lipoprotein – cholesterol) in 23.1% of patients, high LDL-cholesterol (low-density lipoprotein – cholesterol) in 17.4% and high triglycerides in 12.2%. When combining all four cardiometabolic risk factors, only 35.2% of adults and 48.2% of children did not have any risk factor. A total of 61 patients (24.7%) cumulated two or more risk factors. Additionally, metabolic syndrome afflicted 22 participants (8.9%) as per the International Diabetes Federation (IDF) definition and 14 adults (8.6%) using the National Cholesterol Education Program- Adult Treatment Panel (NCEP-ATP) III definition.

**Table 2:** Distribution of cardiometabolic outcomes in survivors of childhood acute lymphoblastic leukemia from the PETALE cohort.

	**Total**	**Adults**	**Men**	**Women**	**Children**	**Boys**	**Girls**
	(N = 247)	(N = 162)	(N = 80)	(N = 82)	(N = 85)	(N = 42)	(N = 43)
	N (%)						
**Obesity**							
Normal	119 (48.2)	71 (43.8)	47 (58.7)	24 (29.3)	48 (56.5)	22 (52.4)	26 (60.5)
Overweight	47 (19.0)	39 (24.1)	19 (23.8)	20 (24.4)	8 (9.4)	4 (9.5)	4 (9.3)
Obesity	81 (32.8)	52 (32.1)	14 (17.50)	38 (46.3)	29 (34.1)	16 (38.1)	13 (30.2)
**Hypertension**							
Normal	217 (87.9)	141 (87.0)	64 (80.0)	77 (93.9)	76 (89.4)	34 (81.0)	42 (97.7)
Pre-/hypertension	30 (12.2)	21 (13.0)	16 (20.0)	5 (6.1)	9 (10.6)	8 (19.0)	1 (2.3)
**Prediabetes**							
Normal	239 (96.8)	155 (95.7)	78 (97.5)	77 (93.9)	84 (98.8)	42 (100.0)	42 (97.7)
Prediabetes	8 (3.2)	7 (4.3)	2 (2.5)	5 (6.1)	1 (1.2)	0 (0.0)	1 (2.3)
**Dyslipidemia**							
Normal	145 (58.7)	84 (51.9)	45 (56.2)	39 (47.6)	61 (71.8)	26 (61.9)	35 (81.4)
Dyslipidemia	102 (41.3)	78 (48.1)	35 (43.8)	43 (52.4)	24 (28.2)	16 (38.1)	8 (18.6)
**Cardiometabolic risk factors**							
0 risk factor	98 (39.7)	57 (35.2)	35 (43.8)	22 (26.8)	41 (48.2)	15 (35.7)	26 (60.5)
1 risk factor	88 (35.6)	61 (37.7)	28 (35.0)	33 (40.2)	27 (31.8)	16 (38.1)	11 (25.6)
2 risk factors	51 (20.7)	36 (22.2)	13 (16.3)	23 (28.1)	15 (17.7)	9 (21.4)	6 (14.0)
3 risk factors	9 (3.6)	7 (4.3)	3 (3.8)	4 (4.9)	2 (2.3)	2 (4.8)	0 (0.0)
4 risk factors	1 (0.4)	1 (0.6)	1 (1.25)	0 (0.0)	0 (0.0)	0 (0.0)	0 (0.0)
**Metabolic syndrome**							
NCEP-ATP III (N = 162)	14 (8.6)	14 (8.6)	5 (6.3)	9 (11.0)	—	—	—
IDF	22 (8.9)	19 (11.7)	9 (11.3)	10 (12.2)	3 (3.6)	3 (7.3)	0 (0.0)

The number of cardiometabolic risk factors was determined by adding the presence of these four factors: obesity/overweight, pre-hypertension/hypertension, insulin resistance and dyslipidemia. NCEP-ATP III: National Cholesterol Education Program - Adult Treatment Panel III; IDF: International Diabetes Federation.

### Predictors of cardiometabolic complications among cALL survivors

Univariate analyses indicated that age at interview (per unit of year) was significantly associated with obesity (Relative risk, RR: 1.03, 95% confidence interval (CI): 1.00–1.06), dyslipidemia (RR: 1.04, 95% CI: 1.02–1.06), prediabetes (RR: 1.11, 95% CI: 1.01–1.22), metabolic syndrome as per the IDF definition (RR: 1.09, 95% CI: 1.03–1.15) and accumulating 2 or more risk factors (RR: 1.06, 95% CI: 1.02–1.09) (Table 3). Male gender was associated with pre-hypertension/hypertension (RR: 4.10, 95% CI: 1.74–9.68) but was protective for obesity (RR: 0.60, 95% CI: 0.41–0.88). Exposure to CRT was only significantly predictive of dyslipidemia (RR: 1.56, 95% CI: 1.11–2.18) (Table 3) and particularly of high LDL-cholesterol (RR: 5.17, 95% CI: 2.11–12.68) (Supplementary Table 3). Being obese at the end of cALL treatment was significantly associated with the presence of obesity at interview (RR: 2.17, 95% CI: 1.47–3.21) and metabolic syndrome – IDF definition (RR: 3.23, 95% CI: 1.28–8.14) (Table 3). However, delta BMI percentile was not a predictor of long-term cardiometabolic complications in our cohort.

**Table 3:** Predictors of cardiometabolic complications in survivors of childhood acute lymphoblastic leukemia: simple log-binomial regression univariate analyses.

	Obesity	Dyslipidemia	Prediabetes	Pre-AHT/AHT	Metabolic syndrome (NCEP)	Metabolic syndrome (IDF)	≥2 Risk factors
	Relative Risk (95% CI)						
CRT (yes vs. none)	1.36 (0.93–2.00)	1.56* (1.11–2.18)	4.76 (0.60–38.11)	1.87 (0.87–4.03)	2.60 (0.60–11.19)	2.33 (0.89–6.11)	1.76* (1.07–2.90)
Gender (males vs. females)	0.60 (0.41–0.88)	1.02 (0.76–1.38)	0.34 (0.07–1.66)	4.10* (1.74–9.68)	0.57 (0.20–1.62)	1.24 (0.56–2.76)	0.87 (0.56–1.35)
Age at diagnosis (per unit of year)	1.02 (0.98–1.06)	1.02 (0.99–1.05)	1.09 (0.96–1.24)	1.02 (0.95–1.09)	1.01 (0.92–1.12)	1.06 (0.98–1.14)	1.02 (0.98–1.07)
Age at interview (per unit of year)	1.03* (1.00–1.06)	1.04* (1.02–1.06)	1.11* (1.01–1.22)	1.01 (0.96–1.06)	1.03 (0.94–1.13)	1.09* (1.03–1.15)	1.06* (1.02–1.09)
Obesity at the end of treatment (yes vs. none)	2.17* (1.47–3.21)	1.09 (0.72–1.65)	1.01 (0.12–8.82)	1.07 (0.42–2.68)	2.33 (0.74–7.35)	3.23* (1.28–8.14)	1.70 (0.98–2.94)
Δ Percentile BMI (unit = 5%)	1.03 (0.98–1.07)	0.98 (0.94–1.01)	1.03 (0.87–1.22)	0.99 (0.91–1.07)	1.06 (0.96–1.18)	1.06 (0.96–1.16)	1.02 (0.97–1.08)

Simple log-binomial regression analysis for each cardiometabolic complication as a function of each predictor was performed. CRT: cranial radiotherapy; BMI: body mass index; AHT: arterial hypertension; IDF: International diabetes Federation; NCEP, National Cholesterol Education Program - Adult Treatment Panel III (NCEP-ATP III); CI: confidence interval. Δ BMI percentile = percentile BMI at end of treatment - percentile BMI at diagnosis. *P < 0.05.

As outlined in Table 4 and Supplementary Table 4, multiple log-binomial regression analyses revealed that CRT exposure remained a predictor of dyslipidemia (RR: 1.60, 95% CI: 1.07–2.41) and high LDL-cholesterol (RR: 4.78, 95% CI: 1.72–13.28) along with age at interview (RR: 1.05, 95% CI: 1.01–1.08 and RR: 1.11, 95% CI 1.04–1.19 respectively). Male gender stayed a predictor for pre- and hypertension (RR: 5.12, 95% CI: 1.81–14.46) in the corresponding adjusted model, but did not remain significant for obesity. Moreover, obesity at the end of treatment was predictor of obesity at interview (RR: 2.07, 95% CI: 1.37–3.14) and metabolic syndrome (IDF definition, RR: 3.04, 95% CI: 1.14–8.09). No associations were found with the variation in BMI percentile between diagnosis and the end of treatment. Of note, metabolic syndrome as defined by the NCEP-ATP III and prediabetes could not be analyzed with multiple log-binomial or Poisson regressions because of convergence problems identified by the SAS procedure PROC GENMOD.

**Table 4:** Predictors of cardiometabolic complications in survivors of childhood acute lymphoblastic leukemia: multiple regression univariate analyses.

	Obesity	Dyslipidemia	Pre-AHT/AHT	Metabolic syndrome (IDF)	≥2 Risk factors
	Relative Risk (95% CI)				
CRT (yes vs. none)	1.30 (0.82–2.05)	1.60* (1.07–2.41)	1.42 (0.61–3.35)	2.45 (0.68–8.76)	1.63 (0.89–3.01)
Gender (males vs. females)	0.66 (0.44–1.00)	1.01 (0.73–1.41)	5.12* (1.81–14.5)	1.56 (0.59–4.07)	1.02 (0.60–1.72)
Age at diagnosis (per unit of year)	1.02 (0.97–1.08)	0.96 (0.92–1.01)	1.04 (0.94–1.15)	1.04 (0.93–1.18)	1.00 (0.93–1.08)
Age at interview (per unit of year)	1.00 (0.96–1.05)	1.05* (1.01–1.08)	0.98 (0.89–1.07)	1.05 (0.96–1.16)	1.02 (0.97–1.08)
Obesity at the end of treatment (yes vs. none)	2.07* (1.37–3.14)	1.05 (0.71–1.55)	1.19 (0.48–2.97)	3.04* (1.14–8.09)	1.55 (0.88–2.73)
Δ Percentile BMI (unit = 5%)	1.02 (0.97–1.06)	0.98 (0.95–1.02)	1.00 (0.93–1.07)	1.06 (0.96–1.17)	1.02 (0.96–1.07)

Multiple log-binomial regression (or Poisson regression with robust variance estimates for obesity) analysis was performed with a model including the predictors: gender, exposure to CRT, age at diagnosis, age at interview, obesity at end of treatment and delta BMI percentile. CRT: cranial radiotherapy; BMI: body mass index; AHT: arterial hypertension; IDF: International diabetes Federation. Δ BMI percentile = percentile BMI at end of treatment - percentile BMI at diagnosis. Metabolic syndrome as defined by the National Cholesterol Education Program - Adult Treatment Panel III (NCEP-ATP III) and prediabetes could not be analyzed because of convergence problems. *P < 0.05.

### Comparison to control cohort

**Figure 1:**
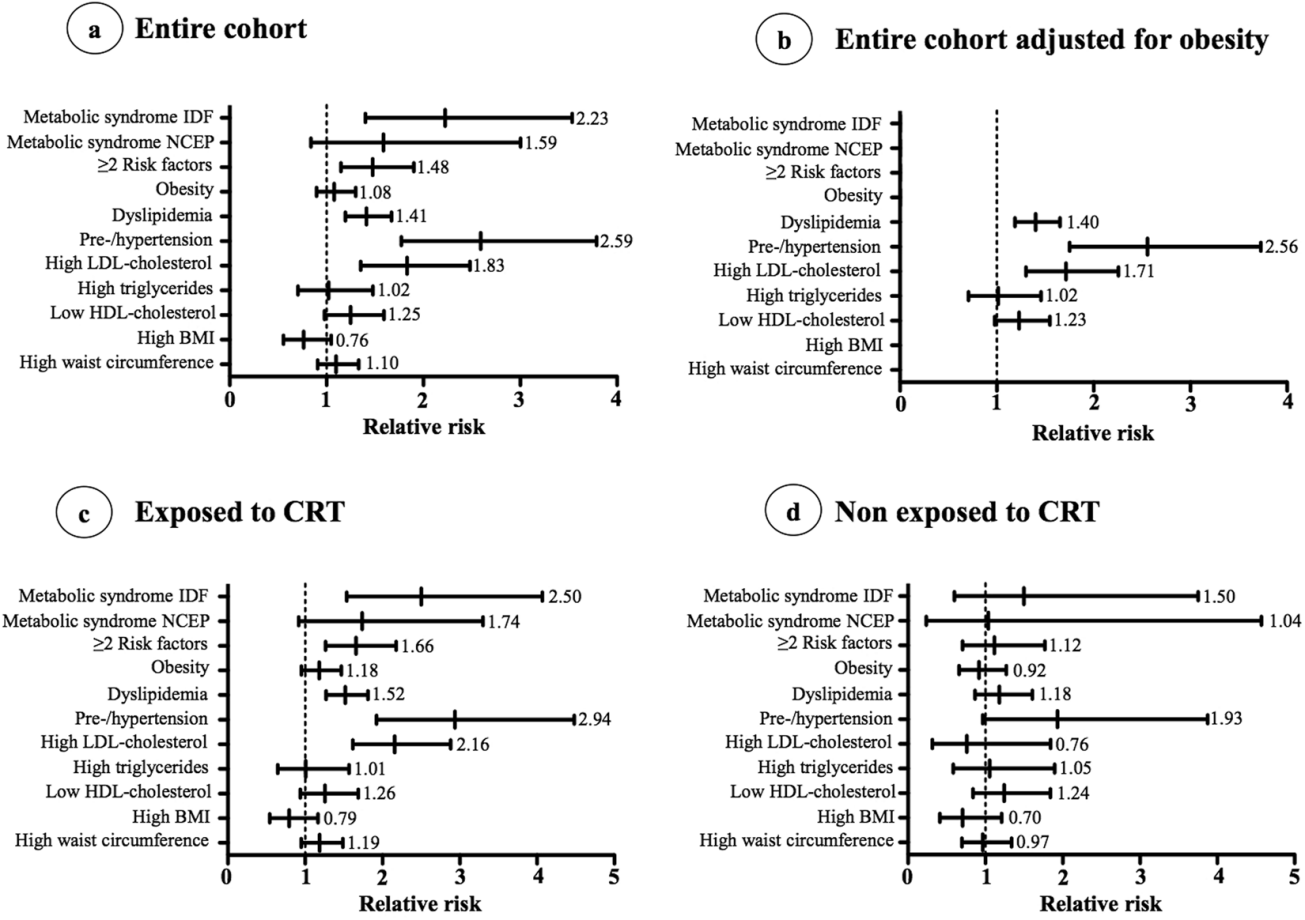
Comparison of cardiometabolic risk factors among PETALE and Canadian Health Measures Survey cohorts. IDF, International Diabetes Federation; NCEP-ATP III, National Cholesterol Education Program - Adult Treatment Panel III; LDL: low-density lipoprotein; HDL, high-density lipoprotein; BMI, body mass index.

Compared to CHMS controls and after adjusting for age and gender, cALL survivors were significantly at higher risk of having the metabolic syndrome (IDF definition), dyslipidemia, pre-hypertension/hypertension while the risk for obesity was not different (Fig. 1a). Survivors were also at higher risk of accumulating 2 or more complications and having high LDL-cholesterol. Adjusting for the presence of obesity hardly modified the RR (Fig. 1b). Stratification according to CRT exposure revealed that only CRT recipients were more likely than CHMS controls to have cardiometabolic complications as the RR were not significant in survivors not exposed to CRT (Fig. 1c,d). However, in participants not exposed to CRT, the relative risk for pre-hypertension and hypertension was close to significance (RR = 1.93, 95% CI 0.96–3.88). Prediabetes, high glucose and glycated hemoglobin (HbA1c) could not be included in the analysis given that the number of cases in the CHMS cohort was too low to respect Statistics Canada confidentiality policies.

## Discussion

One of the main objectives of the PETALE initiative was to characterize early-onset late adverse effects in a homogenous population of CAYA cALL survivors exposed to Dana Farber Cancer Institute protocols from 1987 to 2005. Evaluation of this cohort exposed the high prevalence of cardiometabolic complications and supported an increased cardiovascular risk compared to the general Canadian population, extending the observations obtained by other groups in older cohorts^4–9,20^.

Depending on the definition, we identified the metabolic syndrome in 11.7 and 8.6% of adults (both genders combined) while, in children, 7.3% of boys and no girls were afflicted. These results are similar to that of the French L.E.A. study that has reported an overall metabolic syndrome prevalence of 6.9% (NCEP-ATP III definition) in a relatively young cohort (mean age of 24.2 years)^10^. Furthermore, we found that more than 60% of our cohort presented with at least one cardiometabolic risk factor, similar to what Kourti and al. had found when they investigated 52 survivors from cALL 37 months after the completion of therapy and treated with chemotherapy alone (median age at visit of 15.2 years). In their study, the three criteria for the metabolic syndrome (high triglyceride levels, glucose intolerance, and obesity) were fulfilled by only three subjects (5.76%), while 55.7% had at least one criterion for metabolic syndrome^21^. In the PETALE cohort, the RR for metabolic syndrome (IDF definition) was 2.23 and remained significant only in participants exposed to CRT (RR of 2.50). The RR was not significant when the NCEP-ATP III definition was used, probably because of a smaller cohort size since this definition applies only to adults. The St. Jude Lifetime Cohort Study identified a RR of 1.76 of metabolic syndrome compared to the general population and using the NCEP-ATP III definition^6^. Like us, this higher risk was only significant in those who were exposed to CRT^6^. Saultier and collaborators have found age at interview, male gender and BMI at diagnosis as risk factors for metabolic syndrome and no relation was revealed with CRT exposition, while BMI at end of treatment was not analyzed in this particular study^10^. Finally, an interesting finding of our study is that obesity at the end of treatment was the only predictor of metabolic syndrome that remained significant in the multivariate model, again advocating for early prevention and intervention during treatment in children.

Although our results showed that a large proportion of the PETALE cohort was afflicted with overweight (19.0%) and obesity (32.8%), the risk was not superior compared to the general population even after stratification for CRT exposure. Moreover, being obese did not explain the increased risk of dyslipidemia, pre-hypertension/hypertension or high LDL-cholesterol as adjusting for obesity status did not modify our findings, suggesting that obesity might not be the mechanism explaining their development. In line with our results, a study including very young survivors (median age of 13.3 years) demonstrated that, for standard risk patients, rates of overweight and obesity are not greater than those in non-cancer peers^15^. While we did not find significant associations with the variation of BMI percentile between diagnosis and the end of treatment, obesity at the end of cALL treatment was a strong predictor of obesity in survivors. Similarly, Razzouk *et al*.^22^ observed that young age (<6 years) and overweight/obesity at diagnosis were the best predictors of obesity at adulthood in a study of 456 childhood ALL and lymphoma survivors. That study concluded that BMI weight category at diagnosis, rather than type of central nervous system treatment received, predicted adult weight in long-term survivors of childhood hematologic malignancies. Contrary to us, the authors did not analyze the association with obesity at end of treatment.

The prevalence of dyslipidemia in the PETALE cohort was particularly high, affecting more than 40% of participants. These numbers are superior to another study performed on young cALL survivors (median age of 11.7, range: 7–22) that found 23.3% of 60 patients with dyslipidemia^23^. Our results showed that CRT exposure and older age at interview were predictors of this late effect, specifically for high LDL-cholesterol. Also, cALL survivors were at higher risk of dyslipidemia than the general population, a finding that, after stratification according to CRT, did not remain statistically significant in those who received chemotherapy alone. These high rates and elevated risk of dyslipidemia raise concerns, especially since a study reported that only 5.7% of young cALL survivors were screened for lipids^24^ whereby our findings suggest that they should have a rigorous follow-up. Defining risk factors for dyslipidemia is thus important to improve early screening with frequent, early and appropriate risk-based follow-up for cALL survivors.

Given their young age, the high prevalence (20%) of pre-hypertension and hypertension in male PETALE participants is concerning. We found that male gender, rather than CRT exposure, was strongly predictive of this outcome. Prevalence of hypertension in other studies ranged from 8.3 to 36.7% in which the impact of gender was not mentioned^10,23,25,26^. We also observed an increased risk of pre-hypertension and hypertension compared to the general population that remained significant after controlling for obesity. Similarly to us, the study on the St. Jude Lifetime Cohort found a higher risk compared to controls (RR of 4.23) and for male gender (RR of 1.23)^6^. Results from a French cohort of cALL survivors also reported male sex (Odds ratio, OR = 3.47) and age at last evaluation (OR = 1.08 for each additional year of follow-up) as risk factors for increased arterial pressure^10^. Although the etiology of hypertension in cALL survivors is not fully understood, the developing cardiovascular system of children is thought to be highly susceptible to the toxic effects of chemotherapeutic agents^25^ that could directly or indirectly cause endothelial damage^27,28^. Furthermore, even though we found that the risk of having pre-hypertension or hypertension was higher in survivors exposed to CRT, the RR was close to statistical significance for unexposed participants suggesting that both CRT exposure and chemotherapeutic agents contribute to the risk. Several studies have supported that cALL survivors are at increased risk of type 2 diabetes^6,29,30^. The prevalence of prediabetes was low in our study (n = 8 participants), given the young average age of our cohort, preventing significant associations and causing convergence problems in our multiple models. However, age at interview was marginally associated with prediabetes. Another study showed that in adolescent cancer survivors 5 years or more from diagnosis, anthracyclines, platinum, CRT, and steroids were most strongly associated with insulin resistance^4^. Chow *et al*. demonstrated the development of insulin resistance in pediatric patients undergoing maintenance therapy, as measured prior to and during or soon after a 5-day course of corticosteroids^31^. In our analyses, for the same age range in the CHMS cohort the number of subjects with prediabetes was too little to respect confidentiality policies, indicating the very low prevalence of this outcome in the general population.

ALL treatment may contribute to deteriorate cardiometabolic health through several mechanisms, for example by damaging endocrine organs or afflicting endothelial function and adipose tissue metabolism^32^. CRT has been proposed as a contributing factor to the development cardiometabolic complications^6,13^ and a higher prevalence was reported when chemotherapy is combined with radiotherapy^33^. Our study supported these findings as only survivors exposed to CRT were at higher cardiometabolic risk than the general population. However, other studies indicated that chemotherapy alone could promote these late effects. The prevalence of insulin resistance^30^, obesity^33^ and other cardiovascular risk factors^30^ were found significantly higher in young adult survivors of cALL than in controls, whether or not patients were exposed to CRT. However, in our study, we did not find any relation between CRT and obesity. Similarly, a study from Children’s Oncology Group including 1,638 children enrolled in cALL treatment protocol from 1996 to 2002 revealed that the increased risk of obesity was independent of CRT^34^.

In summary, our study with the PETALE cohort revealed the higher risk of cardiometabolic complications in CAYA cALL survivors. Those who were exposed to CRT were especially at higher risk despite doses of 20 Gy or less. Obesity at the end of treatment was a predictor of obesity at interview and of metabolic syndrome, male gender was predictive of pre-hypertension and hypertension and CRT exposure of dyslipidemia, specifically high LDL-cholesterol. Our results support the need for early lifestyle intervention, preferable during treatment in childhood. Identifying early predictors of cardiometabolic complications will help develop targeted prevention strategies for long-term complications and personalize follow-up.

## Methods

### Aims

There is a need to assess the cardiometabolic risk in populations of CAYA cALL survivors and to identify early predictors for these complications in order to tailor treatments and follow-up for patients most at risk. Hence, the objectives of this study were to: (1) assess the prevalence of obesity, hypertension, insulin resistance, dyslipidemia and metabolic syndrome in CAYA survivors of cALL; (2) identify if exposure to CRT in doses less than 20 Gy, age at diagnosis and obesity status during and after treatment can predict long-term cardiometabolic complications and; (3) evaluate the risk of cardiometabolic risk factors in CAYA cALL survivors as compared to the general Canadian population.

### PETALE Cohort

Participants included in this study were survivors of cALL recruited as part of the PETALE study at Sainte-Justine University Health Center (SJUHC) in Montreal, Canada. This study was designed to characterize long-term effects in cALL survivors^35^. Briefly, subjects enrolled in the PETALE study were treated for cALL at SJUHC with the Dana Farber Cancer Institute protocols^36^. Survivors less than 19 years old at diagnosis, more than 5 years post diagnosis and who have never experienced a relapse were invited to participate. At visit, participants completed a core laboratory assessment as well as anthropometric and clinical evaluations. Participants were residents of the Province of Quebec, Canada and of European descent belonging to a population with an established genetic founder effect^37,38^.

### Comparison Cohort

Controls were selected from Cycle 3 (2012–2013) of the Canadian Health Measures Survey (CHMS). Procedures and methods for data collection for the survey have been described previously^39,40^. This survey, conducted by Statistics Canada, was completed by a sample of the population representing approximately 96.3% of Canadians aged 6 to 79 years. The ethics approval process for the CHMS has been described previously and all participants gave their informed consent^41^. For each metabolic complication, PETALE participants were merged with the Caucasian CHMS controls who had no prior cancer history, were not pregnant, were in the same age range as the PETALE study participants, and for which corresponding clinical measurements where available (565 to 2212 controls depending of the metabolic outcome). Since outcome prevalence between the residents of the Province of Quebec and the residents of Canada (including all provinces) were not statistically significant, analyses were performed using the entire Canadian cohort in order to increase power.

### Outcome measurements

Cut-off values used to determine cardiometabolic outcomes in children (<18 years old) and adults are summarized in Supplementary Table 1. BMI was calculated as weight (kg) / height (m)^2^. For subjects 18 years old and over, BMI ≥25 kg/m^2^ was defined as overweight whereas BMI ≥ 30 kg/m^2^ referred to obesity. For children and adolescents, BMI ≥85^th^ and <97^th^ percentile referred to overweight and ≥97^th^ percentile to obesity according to the World Health Organization BMI charts^42^. For adults, waist circumference was classified as normal (<94 cm in men and <80 cm in women), borderline (≥94 cm and <102 cm in men and ≥80 cm and <88 cm in women)^43^ or high (≥102 cm in men and ≥88 cm in women)^44^. For children, waist circumference below the 90^th^ percentile was defined as normal, ≥90^th^ and <95^th^ percentile as borderline and ≥95^th^ percentile as high^45^. Overall obesity was determined by presenting at least one of two factors: obese according to BMI and/or high waist circumference.

Arterial pressure was measured in the morning on the right arm of the subjects seated and at rest. Hypertension and pre-hypertension were determined when either systolic or diastolic readings were above current recommendations in adults (normal <130/85; pre-hypertension: ≥130/85 and <140/90; hypertension ≥140/90)^46^ and in children (normal: <90^th^ percentile; pre-hypertension: ≥90^th^ and <95^th^ percentile and hypertension ≥95^th^ percentile according to age and height)^47^. Participants currently taking drugs to treat hypertension were also considered hypertensive.

Blood samples were drawn in the morning, after an overnight fasting. Serum was used to assess prediabetes by measuring glucose and glycated hemoglobin (HbA1c). For fasting glucose, values <5.6 mmol/L were considered optimal, ≥5.6 and <6.1 mmol/L were considered at risk and ≥6.1 and <7.0 mmol/L indicated prediabetes and ≥7.0 mmol/L diabetes^48^. For HbA1c, values <5.5% (<37 mmol/mol) were optimal, between 5.5 and 5.9% (37 and 41 mmol/mol) at risk and between 6.0% and 6.4% (42 and 46 mmol/mol) referred to prediabetes^48^. Overall prediabetes was defined as presenting at least one of two factors: blood fasting glucose ≥6.1 mmol/L and/or HbA1c ≥6.0% (42 mmol/mol)^48^.

Dyslipidemia was evaluated in fasting serum. In adults, low-density lipoprotein (LDL)-cholesterol <2.6 mmol/L was considered optimal, ≥2.6 and <3.4 mmol/L borderline and ≥3.4 mmol/L high^49^. Triglycerides levels <1.3 mmol/L were classified optimal, ≥1.3 and <1.7 mmol/L borderline^50^ and ≥1.7 mmol/L high^49^. High-density lipoprotein (HDL)-cholesterol <1.03 in men and <1.3 mmol/L in women were considered low^49^. In children, LDL-cholesterol, triglycerides and HDL-cholesterol values were classified according to the recent guidelines of the National Heart, Lung and Blood Institute for gender and age group and were classified as optimal, borderline or abnormal^50^. Dyslipidemia was determined by having at least one factor: high LDL-cholesterol; high triglycerides; low HDL-cholesterol and/or on drug treatment.

As per the National Cholesterol Education Program- Adult Treatment Panel (NCEP-ATP) criteria^51^, metabolic syndrome was identified as presence of three or more of the following: (i) waist circumference ≥102 cm in men and ≥88 cm in women; (ii) hypertriglyceridaemia ≥1.70 mmol/L or on drug treatment; (iii) low HDL <1.03 mmol/L in men and <1.3 mmol/L in women or on therapy; (iv) systolic blood pressure ≥130 mmHg or diastolic ≥85 mmHg or on treatment and; (v) hyperglycaemia ≥5.55 mmol/L or on treatment. According to the International Diabetes Federation (IDF)^52^, metabolic syndrome was defined in participants 16 years and older having waist circumference ≥94 cm in men and ≥80 cm in women, plus any two of the following factors: (i) triglycerides ≥1.70 mmol/L or on drug treatment; (ii) HDL <1.03 mmol/L in men and <1.3 mmol/L in women or on therapy; (iii) systolic ≥130 mmHg or diastolic ≥85 mmHg or on treatment and; (iv) fasting blood glucose ≥5.6 mmol/L. For children 10 to <16 years old, metabolic syndrome (IDF) was defined as waist circumference ≥90^th^ percentile plus any two of: (i) triglycerides ≥1.70 mmol/L; (ii) HDL <1.03 mmol/L; (iii) systolic ≥130 mmHg or diastolic ≥85 mmHg and; (iv) fasting blood glucose ≥5.6 mmol/L.

The cumulative number of cardiometabolic risk factors was determined by adding the presence of these four factors: obesity, pre-hypertension/hypertension, prediabetes and dyslipidemia.

### Measurements of overweight, obesity and body mass index during treatments

Data on weight and height at diagnosis and at end of treatment were used to assess BMI percentiles according to age and gender. Presence of overweight and obesity was evaluated using the World Health Organization BMI charts as described above^42^. Delta BMI percentile was calculated as [Δ BMI percentile = percentile BMI at end of treatment - percentile BMI at diagnosis].

### Statistical analyses

Descriptive statistics were used to report the characteristics of the cALL survivors and the distribution of cardiometabolic outcomes in the PETALE cohort. In our study, the prevalence of many outcome measures was larger than 15%. Since odds ratio (OR) cannot be interpreted as a RR when outcome is not rare^53^, we used log-binomial regression models for the estimation of RR (both unadjusted and adjusted) and corresponding 95% confidence intervals (CI). We first performed a series of simple log-binomial regression analyses to assess the association between each cardiometabolic complication and each targeted potential predictors: gender, age at interview, age at diagnosis, exposure to CRT, obesity at diagnosis, obesity at the end of treatment and delta BMI percentile. A multiple log-binomial regression analysis using the same set of predictors was then performed for each outcome. Poisson regression with robust variance estimates was applied in the case of non-convergent log-binomial models^54,55^.

To estimate the RR and 95% CI of having a particular cardiometabolic complication among cALL survivors versus CHMS controls, we performed log-binomial regression or Poisson regression with robust variance estimates analysis^54^ with adjustment for sex and age at visit. Secondary stratified analyses comparing PETALE sub-cohorts defined by exposure to CRT (exposed vs. unexposed) to CHMS controls were subsequently executed. To assess whether being cALL survivor is associated with the outcomes independently of obesity status, we then adjusted each model for presence of obesity at interview. Analyses were performed using SAS 9.4 and SAS Studio. A P-value <0.05 was considered significant.

### Availability of data and materials

The datasets generated and/or analysed during the current study are not publicly available due to confidentiality reasons but are available from the corresponding author on reasonable request.

### Ethics approval and consent to participate

The study was approved by the Institutional Review Board of SJUHC and investigations were carried out in accordance with the principles of the Declaration of Helsinki. Written informed consent was obtained from study participants or parents/guardians.

## Electronic supplementary material

Supplementary Information
