# Supplementary material for: Cardiometabolic Risk Factors in Childhood, Adolescent and Young Adult Survivors of Acute Lymphoblastic Leukemia – A Petale Cohort
Source: Sci Rep. 2017 Dec 15;7:17684. doi: 10.1038/s41598-017-17716-0 (PMC5732194; doi:10.1038/s41598-017-17716-0)
Supplement: Supplementary file 1 — Supplementary Information [file 41598_2017_17716_MOESM1_ESM.pdf]

Supplementary Information

CARDIOMETABOLIC RISK FACTORS IN CHILDHOOD, ADOLESCENT AND YOUNG ADULT SURVIVORS OF ACUTE LYMPHOBLASTIC LEUKEMIA SURVIVORS – A PETALE COHORT

Emile Levy[1], Mariia Samoilenko[3], Sophia Morel[1], Jade England[2], Devendra Amre[2], Laurence Bertout[2], Simon Drouin[2], Caroline Laverdière[2], Maja Krajinovic[2], Daniel Sinnett[2], Geneviève Lefebvre[3], Valérie Marcil[1*]

Research Centre, Sainte-Justine University Health Center, Departments of [1]Nutrition and [2]Pediatrics, Université de Montréal, Montreal, Quebec, Canada, H3T 1C5, [3]Department of Mathematics, Université du Québec à Montréal, Montreal, Quebec, Canada, H3C 3P8.

Supplementary Table 1. Cut-off values for cardiometabolic outcomes

Risk factor	Adults	Children
**BMI**		
Overweight	>25 and <30 kg/m^2	>85th and <97th percentile
Obesity	≥30 kg/m^2	≥97th percentile
**Waist circumference**		
Borderline	≥94 and <102 cm (men); ≥80 and <88 cm (women)	≥90th and <95th percentile
High	≥102 cm (men) and ≥88 cm (women)	≥95th percentile
**Hypertension**		
Pre-hypertension	≥130/85 and <140/90 mmHg	≥90th and <95th percentile for age and height
Hypertension	≥140/90 mmHg	≥95th percentile for age and height
**Blood fasting glucose**		
At risk	≥5.6 and <6.1 mmol/L	≥5.6 and <6.1 mmol/L
Prediabetes	≥6.1 mmol/L	≥6.1 mmol/L
**Glycated hemoglobin**		
At risk	≥5.5 and <6%	≥5.5 and <6%
Prediabetes	≥6% and <6.5%	≥6% and <6.5%
**Triglycerides**		
Borderline	≥1.3 and <1.7 mmol/L	≥1.00 and <1.47 mmol/L
High	≥1.7 mmol/L	≥1.47 mmol/L
**LDL-cholesterol**		
Borderline	≥2.6 and <3.4 mmol/L	≥2.85 and <3.36 mmol/L
High	≥3.4 mmol/L	≥3.36 mmol/L
**HDL-cholesterol**		
Low	<1.03 (men) and <1.3 mmol/L (women)	<1.03 mmol/L

BMI, body mass index; HOMA-IR, homeostasis model assessment; LDL, low-density lipoprotein; HDL, high-density lipoprotein.

Supplementary Table 2. Distribution of cardiometabolic outcomes according to cut-off values of survivors of childhood acute lymphoblastic leukemia from the PETALE cohort

	**Total** (N=247)	**Adults** (N=162)	**Men** (N=80)	**Women** (N=82) **N (%)**	**Children** (N=85)	**Boys** (N=42)	**Girls** (N=43)
**BMI**							
Normal	152 (61.5)	92 (56.8)	49 (61.2)	43 (52.4)	60 (70.6)	26 (61.9)	34 (79.1)
Overweight	60 (24.3)	42 (25.9)	21 (25.3)	21 (25.6)	18 (21.2)	11 (26.2)	7 (16.2)
Obesity	35 (14.2)	28 (17.3)	10 (12.50)	18 (22.0)	7 (8.2)	5 (11.9)	2 (4.7)
**Waist circumference**							
Normal	131 (53.0)	80 (50.0)	56 (70.00)	25 (30.5)	50 (58.8)	24 (57.1)	26 (60.5)
Borderline	38 (15.4)	32 (19.7)	13 (16.2)	19 (23.2)	6 (7.1)	2 (4.8)	4 (9.3)
High	78 (31.6)	49 (30.3)	11 (13.8)	38 (46.3)	29 (34.1)	16 (38.1)	13 (30.2)
**Systolic blood pressure**							
Normal	222 (89.9)	145 (89.5)	66 (82.5)	79 (96.3)	77 (90.6)	35 (83.3)	42 (97.7)
Pre-hypertension	21 (8.5)	14 (8.6)	11 (13.7)	3 (3.7)	7 (8.2)	6 (14.3)	1 (2.3)
Hypertension	4 (1.6)	3 (1.9)	3 (3.8)	0 (0)	1 (1.2)	1 (2.4)	0 (0)
**Diastolic Blood pressure**							
Normal	239 (96.8)	155 (95.7)	76 (95.0)	79 (96.3)	84 (98.8)	41 (97.6)	43 (100)
Pre-hypertension	7 (2.8)	6 (3.7)	3 (3.7)	3 (3.7)	1 (1.2)	1 (2.4)	0 (0)
Hypertension	1 (0.4)	1 (0.6)	1 (1.3)	0 (0)	0 (0)	0 (0)	0 (0)
**Fasting blood glucose**							
Optimal	217 (87.9)	136 (84.0)	63 (78.7)	73 (89.0)	81 (95.3)	39 (92.9)	42 (97.7)
At risk	23 (9.3)	19 (11.7)	15 (18.8)	4 (4.9)	4 (4.7)	3 (7.1)	1 (2.3)
Prediabetes	7 (2.8)	7 (4.3)	2 (2.5)	5 (6.1)	0 (0.0)	0 (0.0)	0 (0.0)
**HbA1c**							
Optimal	210 (85.0)	142 (87.6)	71 (88.8)	71 (86.6)	68 (80.0)	33 (78.6)	35 (81.4)
At-risk	33 (13.4)	17 (10.5)	8 (10.0)	9 (11.0)	16 (18.8)	9 (21.4)	7 (16.3)
Prediabetes	4 (1.6)	3 (1.9)	1 (1.2)	2 (2.4)	1 (1.2)	0 (0.00)	1 (2.3)
**HOMA-IR**							
Normal	207 (83.8)	133 (82.1)	69 (86.2)	64 (78.0)	74 (87.1)	37 (88.10)	37 (86.0)
High	40 (16.2)	29 (17.9)	11 (13.8)	18 (22.0)	11 (12.9)	5 (11.90)	6 (14.0)
**Triglycerides**							
Optimal	175 (70.8)	120 (74.0)	64 (80.0)	56 (68.3)	55 (64.7)	27 (64.3)	28 (65.1)
Borderline	42 (17.0)	21 (13.0)	6 (7.5)	15 (18.3)	21 (24.7)	12 (28.6)	9 (20.9)
High	30 (12.2)	21 (13.0)	10 (12.5)	11 (13.4)	9 (10.6)	3 (7.1)	6 (14.0)
**LDL-cholesterol**							
Optimal	139 (56.3)	73 (45.1)	33 (41.2)	40 (48.8)	66 (77.7)	34 (81.0)	32 (74.4)
Borderline	65 (26.3)	53 (32.7)	27 (33.8)	26 (31.7)	12 (14.1)	4 (9.5)	8 (18.6)
High	43 (17.4)	36 (22.2)	20 (25.00)	16 (19.5)	7 (8.2)	4 (9.5)	3 (7.0)
**HDL-cholesterol**							
Optimal	190 (76.9)	119 (73.5)	66 (82.5)	53 (64.6)	71 (83.5)	31 (73.8)	40 (93.0)
Low	57 (23.1)	43 (26.5)	14 (17.5)	29 (35.4)	14 (16.5)	11 (26.2)	3 (7.0)

BMI, body mass index; HOMA-IR, homeostasis model assessment; LDL, low-density lipoprotein; HDL, high-density lipoprotein; HbA1c: glycated hemoglobin.

Supplementary Table 3. Predictors of cardiometabolic complications in survivors of childhood acute lymphoblastic leukemia: simple log-binomial regression univariate analyses

	High BMI	High waist circumference	High TG	High LDL-cholesterol	Low HDL-cholesterol	High glucose	High HbA1c
	Relative Risk (95% CI)						
CRT (yes vs. none)	1.30<br>(0.68-2.50)	1.29<br>(0.87-1.90)	1.18<br>(0.58-2.36)	5.17*<br>(2.11-12.68)	1.17<br>(0.73-1.87)	4.08<br>(0.50-33.39)	2.04<br>(0.22-19.34)
Gender (males vs. females)	0.77<br>(0.41-1.43)	0.54<br>(0.37-0.80)	0.78<br>(0.40-1.54)	1.29<br>(0.75-2.24)	0.80<br>(0.51-1.27)	0.41<br>(0.08-2.07)	0.34<br>(0.04-3.24)
Age at diagnosis<br>(per unit of year)	1.01<br>(0.95-1.08)	1.01<br>(0.97-1.05)	1.05<br>(0.98-1.12)	0.98<br>(0.92-1.05)	1.01<br>(0.96-1.06)	1.13<br>(0.98-1.29)	1.04<br>(0.86-1.27)
Age at interview<br>(per unit of year)	1.08*<br>(1.03-1.12)	1.02<br>(0.99-1.05)	1.04<br>(0.99-1.09)	1.06*<br>(1.02-1.09)	1.03<br>(0.99-1.06)	1.14*<br>(1.03-1.26)	1.08<br>(0.94-1.24)
Obesity at the end of treatment (yes vs. none)	2.90*<br>(1.40-5.54)	2.23*<br>(1.50-3.30)	1.93<br>(0.78-4.02)	0.84<br>(0.37-1.88)	1.09<br>(0.60-2.01)	CP	4.05<br>(0.27-63.37)
Δ Percentile BMI (unit=5%)	1.01<br>(0.94-1.08)	1.03<br>(0.99-1.07)	0.96<br>(0.89-1.04)	0.96<br>(0.90-1.03)	0.99<br>(0.94-1.04)	1.07<br>(0.89-1.30)	0.90<br>(0.68-1.19)

Simple log-binomial regression analysis for each cardiometabolic complication as a function of each predictor was performed. CRT: cranial radiotherapy; BMI: body mass index; RR: relative risk; CI: confidence interval; TG: triglycerides; LDL: low-density lipoprotein; HDL: high-density lipoprotein. Δ BMI percentile = percentile BMI at end of treatment - percentile BMI at diagnosis. CP: This analysis could not be performed because of convergence problems. *P<0.05.

Supplementary Table 4. Predictors of cardiometabolic complications in survivors of childhood acute lymphoblastic leukemia: multiple log-binomial univariate analyses

	High BMI	High waist circumference	High TG	High LDL-cholesterol	Low HDL-cholesterol
	Relative Risk (95% CI)				
CRT (yes vs. none)	1.07 (0.49-2.36)	1.36 (0.87-2.15)	1.29 (0.55-3.01)	4.78* (1.72-13.28)	1.22 (0.70, 2.13)
Gender (males vs. females)	0.95 (0.48-1.90)	0.65 (0.41-1.03)	0.80 (0.37-1.75)	1.12 (0.64-1.94)	0.80 (0.48, 1.33)
Age at diagnosis (per unit of year)	0.97 (0.88-1.07)	1.04 (0.99-1.11)	1.06 (0.96-1.17)	0.87 (0.81-0.95)	0.97 (0.90, 1.05)
Age at interview (per unit of year)	1.11* (1.02-1.20)	1.00 (0.95-1.05)	0.98 (0.91-1.07)	1.11* (1.04-1.19)	1.05 (0.99, 1.10)
Obesity at the end of treatment (yes vs. none)	2.73* (1.32-5.63)	2.14* (1.36-3.38)	1.94 (0.84-4.49)	0.74 (0.34-1.58)	1.00 (0.53, 1.88)
Δ Percentile BMI (unit=5%)	0.99 (0.92-1.06)	1.01 (0.97-1.05)	0.97 (0.89-1.05)	0.96 (0.90-1.01)	0.99 (0.94, 1.04)

Multiple log-binomial regression analysis was performed with a model including the predictors: gender, exposure to CRT, age at diagnosis, age at interview, obesity at end of treatment and delta BMI percentile. CRT: cranial radiotherapy; BMI: body mass index; RR; CI: confidence interval; TG: triglycerides; LDL: low-density lipoprotein; HDL: high-density lipoprotein. Δ BMI percentile = percentile BMI at end of treatment - percentile BMI at diagnosis. *P<0.05.
